# Using phylogeny and functional traits for assessing community assembly along environmental gradients: A deterministic process driven by elevation

**DOI:** 10.1002/ece3.3068

**Published:** 2017-06-02

**Authors:** Jinshi Xu, Yu Chen, Lixia Zhang, Yongfu Chai, Mao Wang, Yaoxin Guo, Ting Li, Ming Yue

**Affiliations:** ^1^ Key Laboratory of Resource Biology and Biotechnology in Western China (Northwest University) Ministry of Education Xi'an China; ^2^ School of Life Sciences Northwest University Xi'an China; ^3^ College of Grassland and Environment Sciences Xinjiang Agricultural University Urumchi China

**Keywords:** community assembly, environmental gradients, functional traits, phylogenetic signal, phylogeny

## Abstract

Community assembly processes is the primary focus of community ecology. Using phylogenetic‐based and functional trait‐based methods jointly to explore these processes along environmental gradients are useful ways to explain the change of assembly mechanisms under changing world. Our study combined these methods to test assembly processes in wide range gradients of elevation and other habitat environmental factors. We collected our data at 40 plots in Taibai Mountain, China, with more than 2,300 m altitude difference in study area and then measured traits and environmental factors. Variance partitioning was used to distinguish the main environment factors leading to phylogeny and traits change among 40 plots. Principal component analysis (PCA) was applied to colligate other environment factors. Community assembly patterns along environmental gradients based on phylogenetic and functional methods were studied for exploring assembly mechanisms. Phylogenetic signal was calculated for each community along environmental gradients in order to detect the variation of trait performance on phylogeny. Elevation showed a better explanatory power than other environment factors for phylogenetic and most traits’ variance. Phylogenetic and several functional structure clustered at high elevation while some conserved traits overdispersed. Convergent tendency which might be caused by filtering or competition along elevation was detected based on functional traits. Leaf dry matter content (LDMC) and leaf nitrogen content along PCA 1 axis showed conflicting patterns comparing to patterns showed on elevation. LDMC exhibited the strongest phylogenetic signal. Only the phylogenetic signal of maximum plant height showed explicable change along environmental gradients. *Synthesis*. Elevation is the best environment factors for predicting phylogeny and traits change. Plant's phylogenetic and some functional structures show environmental filtering in alpine region while it shows different assembly processes in middle‐ and low‐altitude region by different trait/phylogeny. The results highlight deterministic processes dominate community assembly in large‐scale environmental gradients. Performance of phylogeny and traits along gradients may be independent with each other. The novel method for calculating functional structure which we used in this study and the focus of phylogenetic signal change along gradients may provide more useful ways to detect community assembly mechanisms.

## 1 INTRODUCTION

Community assembly has been focused on providing a conceptual foundation for understanding the processes of plant colonization in locality (Chase, 2003). Recently, some community assembly theories have been proposed to detect the patterns of species diversity and distributions at community level (Diamond, 1975). There are two main processes of assembly: Neutral process emphasizes the great importance of random genetic drift of species rather than influence of environment (Kimura, 1991). In contrast, deterministic process deems that the pattern of which and how many species live in community is closely related to abiotic and biotic environment, and may change along the environmental gradients (Cornwell & Ackerly, 2009). In other words, deterministic process is niche‐based.

Previous studies considered that neutral and deterministic processes in conjunction lead to community assembly (Kraft, Valencia, & Ackerly, 2008; Kembel, 2009; Swenson & Enquist, 2009), while deterministic process is the main process during assembly (Purschke et al. 2013; Yang et al., 2014) in particular in temperate region (Wang et al., 2013). Limiting similarity and environmental filtering are two contrary niche‐based mechanisms occur simultaneously along various environmental axes during deterministic assembly even within a single community, and influence the community structure (Weiher & Keddy, 1998; Kraft, 2008; Cornwell & Ackerly, 2010; Mason, Bello, Doležal, & Leps, 2011). In general, limiting similarity is expected to exclude similar species of coexisting species whereas environmental filtering is expected to select much similar species among coexisting species that share similar habitat conditions (Andersen, Endara, Turner, & Dalling, 2012). Nevertheless, it is still a major challenge for ecologists about understanding what and how environment factors drive balance of these processes (Grime, 2006; Mayfield & Levine, 2010; Luo et al., 2016). An increasing suggestion nowadays to merge functional traits and phylogenetic biology has developed the understanding of this challenge (Kraft & Ackerly, 2010), as phenotypic or phylogenetic structure will express some patterns (Kraft, Cornwell, Webb, & Ackerly, 2007). Both trait‐based and community phylogenetic structure approaches provide powerful tools to explore the relative importance of above assembly processes (Webb, Ackerly, Mcpeek, & Donoghue, 2003; Kraft et al., 2008; Kembel, 2009; Swenson & Enquist, 2009; Satdichanh, Millet, Heinimann, Nanthavong, & Harrison, 2015).

Classically, closely phylogenetically related species are more functionally similar than distantly related species (Swenson, Enquist, Thompson, & Zimmerman, 2007). Hence, limiting similarity leads to the divergence of trait values or phylogenetic distance among coexisting species in order to competing with each other for finite resource. In contrast, environmental filtering causing convergence pattern in trait values or phylogenetic distance as some species will be filtered out from some unfit communities, resulting in a reduction in the range of functional traits distribution (Diaz, Cabido, & Casanoves, 1998; Weiher & Keddy, 1998; Kraft et al., 2008; Golodets, Sternberg, & Kigel, 2009; Cornwell & Ackerly, 2009). It should be noted that conclusions reached by trait‐based approach is not always similar to phylogenetic conclusions (Losos, 2008). Actually, phylogeny of species in community represents just conserved traits but not all traits while deterministic processes may also have detectable signature in some plastic traits (Keddy, 1992; Zobel, 1997; Pavoine & Bonsall, 2010; Götzenberger et al., 2012). In previous studies, phylogeny was regarded as alternative of plant functional traits, which are difficult to measure, but not quite good alternative (Gerhold, Cahill, Winter, Bartish, & Prinzing, 2015; Carlucci, Hidasineto, Brum, & Cianciaruso, 2015). There has been a consistent argument that how environment (**E**), phylogeny (**P**), and traits (**T**) interact with each other. One possibility is environment acts on phylogeny firstly and then influences traits (**E → P → T**). The other possibility is environment, and phylogeny influences traits independently (**E → T ← P**) (Yang, Powell, Zhang, & Du, 2012), indicates phylogeny may not has coherent direction with different traits along different environmental gradients.

To achieve mechanistic insight into community assembly processes along environmental gradients, researchers are expected to analyze the phylogenetic composition (Swenson, 2011) and functional traits range synchronously. Phylogeny is a reflection of species evolutionary history on structure of community (Webb 2002; Kembel & Hubbell, 2006) and can help to reveal the multitude of processes driving community assembly in various habitats (Cavender‐Bares, Kozak, Fine, & Kembel, 2009; Cavender‐Bares et al., 2009). Meanwhile, functional traits reflect different evolutionary responses to environmental variation and interaction between species (Wright, Reich, & Westoby, 2001; Donovan, Maherali, Caruso, Huber, & Kroon, 2011), and play an important role in understanding the plant strategies related to resource acquisition, regeneration ability or shade tolerance (Goldberg, 1996; Westoby, Falster, Moles, And, & Wright, 2002; Chave et al., 2009) across environmental gradients (Diaz & Cabido, 1997; Mcgill, Enquist, Weiher, & Westoby, 2006; Cornelissen, Lavorel, Garnier, Díaz, & Gurvich, 2003; Ackerly, 2004; Cornwell & Ackerly, 2009; Hulshof & Swenson, 2010).

As abiotic and biotic filters constrain species establishment via selection on their phylogenetic patterns or functional traits affected by environment (Lebrija‐Trejos, Meave, Bongers, & Poorter, 2010), it is indispensable to detect the relationship among phylogeny, functional traits and environment. Generally speaking, filtering process caused by environmental stress is much more and easier to detect than limiting similarity (Luo et al., 2016). Study on tropical rain forest announced phylogenetic clustering in high disturbance habitats but evenness in other habitats (Ding, Zang, Letcher, Liu, & He, 2012), similar to the study on a seasonal tropical forest which found phylogenetic clustering in sander soil and lower pH causing least favorable to plant growth (Satdichanh et al., 2015). However, there was no evidence about clustering or divergence of traits in both studies. Verdú et al. showed high fire frequency leads to phylogenetic clustering (Verdú & Pausas, 2007), caused by environmental filtering similarly. Moreover, the similar pattern was even showed in avian studies that a hasher habitat results in phylogenetic clustering (Gianuca, Dias, Debastiani, & Leandro, 2014; González‐Caro, Parra, Graham, Mcguire, & Cadena, 2012). By traits studies, filtering process was also detected at early of succession (Radika, Dawson, & Patricia, 2014) and high elevation (Hulshof et al., 2013; Pottier et al., 2012) that represents harsh environment for plant. Hence, deterministic processes in particular filtering process may play a greater role under a harsher environment (Qian, Hao, & Zhang, 2014) even a general environment (Myers et al., 2013; Lasky, Sun, Su, Chen, & Keitt, 2013; Fortunel, Paine, Fine, Kraft, & Baraloto, 2014; Siefert, Ravenscroft, Weiser, & Swenson, 2013; de Bello et al., 2012). However, combining phylogenetic‐based and trait‐based approaches to test community assembly processes along environmental gradients have seldom been applied to the same community although it is necessary to clarify the mystery of community ecology (Kraft & Ackerly, 2010; Satdichanh et al., 2015). In particular, the consistency of performance of community assembly patterns along the same gradient through different methods is still a mystery, while it may reveal some key points of community assembly processes.

Elevational gradient is excellent system for ecologists due to their steeper environmental gradients over short geographical distance (Qian et al., 2014). As shown in the previous example about trait‐based studies, filtering may effort increasing with severity at higher elevation, but the relationship between phylogeny and elevation remains seldom to be researched (Qian et al., 2014). Also, elevational gradients can provide evidence for community responses to long‐term climate changes, and help understand the future of biodiversity in a changing world (Fukami, Bezemer, Mortimer, & Putten, 2005; McCain & Colwell, 2011). Likewise, locality gradients such as soil pH, soil water content, topographic pattern, soil nutrient availability, and even coverage of canopy may influence the trait values and phylogenetic structure as well (Weiher et al., 1999; Bernard‐Verdier et al., 2012; Heineman, Turner, & Dalling, 2016; Luo et al., 2016; John et al., 2007). Meanwhile, some soil characters would be affected by elevation (Lovett & Kinsman, 1990). Howbeit, previous study either focused on single environment factor or experimented on gradients with relative narrow range. There are quite few researches on assembly pattern across multiple large range gradients (including elevation and soil characters). In particular, for phylogenetic signal, change may provide information about the relationship of phylogeny and functional traits along these environmental gradients, yet previous studies only focused on phylogenetic signal as a total index of whole study site.

In this study, we used phylogenetic‐based and trait‐based methods to analyze the variation of community structure along crucial elevation and other locality gradients in the most magnificent mountain of eastern China. Primary aims of this article were to (1) find out environment factors how to influence the functional traits and phylogeny of coexistence species in community, (2) understand how community assembly processes to change along changing environment and (3) prove whether phylogenetic‐based and trait‐based assembly patterns are covariant along environmental gradients and analyze the reason. In order to achieve this goal, we launched experiment by sampling 148 woody species from 40 plots located in different locations along environmental gradients, because dominant growth form species have more detectable processes in community assembly (Gallagher & Leishman, 2012; Webb, Ackerly, & Kembel, 2008). We analyzed the data using variance partitioning to detect main environment factor affecting community, phylogenetic, and functional trait structure based on null model test to discuss assembly patterns along environmental gradients, and phylogenetic signal analysis to locate which ecological similarity between species is related to phylogenetic relatedness along environmental gradients (Losos, 2008).

## 2 MATERIALS AND METHODS

### 2.1 Study site

**Figure 1:**
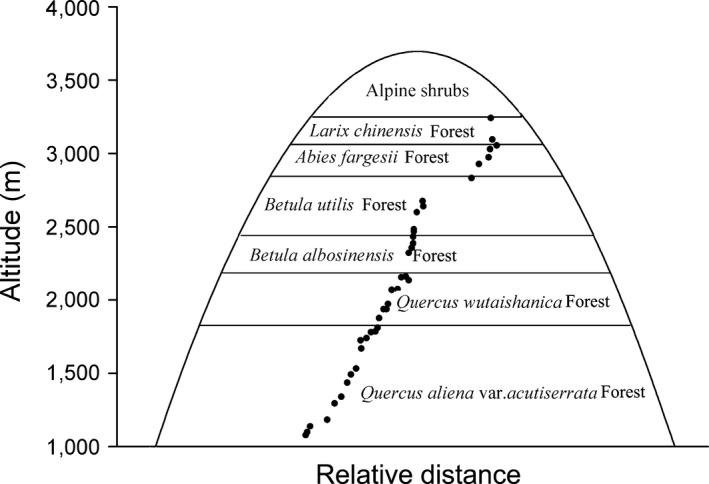
Example of sampling plots. Zonal zone in graph represents the range of this forest type labeled by words. Black plots in graph represent our sampling plots, and relative position of plots on abscissa means the relative distance among plots. Position of plots on ordinate represents the elevation of each plot

The study was carried on a fully protected region in Taibai Mountain Nature Reserve, central China, located on 33°59′45″N–34°05′12″N, 107°41′18″E–107°48′22″E. The reserve has been established since 1965 and is one of the earliest Nature Reserve in China. No fire or human disturbance events happened after that. Taibai Mountain is the highest mountain in the mainland China east of Qinghai–Tibet Plateau, the highest peak of Taibai Mountain is 3,767.2 m. In our study, the range of elevation is 1,140–3,480 m, means that contain over 2,300 m altitude gradient. This region is influenced by continental monsoon, mean annual temperature in our study site varies from 0.9°C to 12.3°C related to elevation (Tang & Fang, 2004), and annual precipitation is 640–1,000 mm, with a hump‐shaped pattern that maximum precipitation occurs at 1,900 m. Forest coverage is over 82%, with a relative high species diversity. In our study region, there are 389 woody species existing potentially recorded by literature, the vegetation distributes along some zonal zone in Taibai Mountain. In other words, vegetation types change along elevation gradient (Zhu, 1981) because of difference of hydrothermal conditions,. The dominant species of each vegetation zone see Figure 1.

### 2.2 Plot set and environment gradients

Forty 20 m × 30 m plots were selected along the elevation gradient from 1,140 to 3,480 m (Table 1) as to gain adequate samplings relative to the study region (Yang et al., 2014). In order to represent the whole study region integrally, we ensure that more than three plots per 200 m altitude range were set as far as possible. Location of each plot was randomly selected, whereas the topographic factors or species composition must different for each plot in 200 m range to maximize variation of environment factors. Owing to the drastic hydrothermal shift caused by elevation changing under a short geographical distance, although elevation ranges from 1,140 to 3,480 m, the maximum geographical distance between pairwise plots is only 13 km, a quite small range for Taibai Mountain region.

**Table 1:** The semimatrix of correlations among environment factors

Factors	Elevation	SWC	TN	AN	NN	RAP	pH	Slope
SWC	0.895**							
TN	0.470**	0.576**						
AN	0.456**	0.582**	0.996**					
NN	−0.005	−0.238	−0.261	−0.351*				
RAP	0.492**	0.548**	0.564**	0.562**	−0.155			
pH	0.349*	0.249	−0.126	−0.170	0.484**	0.056		
Slope	−0.131	−0.279	−0.192	−0.198	0.127	−0.310*	−0.169	
WCD	−0.211	−0.063	0.128	0.137	−0.127	0.229	0.036	−0.260

Factor codes are as follows: elevation, altitude of plot; SWC, soil water content; TN, soil total nitrogen content; AN, soil ammonium nitrogen content; NN, soil nitrate nitrogen content; RAP, soil rapid available phosphorus content; pH, soil pH value; slope: slope of plot; WCD, woody species coverage degree.

*NOVA 0.01 < *p* ≤ .05.

**ANOVA *p* ≤ .01.

All woody species within each plot was identified, the abundance and coverage of each species in plots were documented. We recorded environmental information of each plots, altitude, slope, woody species canopy coverage degree (WCD) should be measured and recorded accurately. Slope and WCD were shown to have correlation with species leaf traits and height (Ackerly & Cornwell, 2007). Altitude was obtained by GPS recorder (HOLUX EZ‐Tour, HOLUX Technology Inc.), and slope was read from compass with slope indicator. We calculated WCD by summating the coverage of species occurring at each plot. Besides, we conducted the soil sampling from each plot with three replicates, each of replicates was obtained from both ends and central point along diagonal of plots, dug out from 10 to 20 cm below ground as characters of this soil layer were relatively stable. After sampling, all soil samples was weighed for soil fresh weight (SW_fresh_) and then air‐dried and weighed again for soil dry weight (SW_dry_). Soil water content (SWC) was calculated a

$$\text{SWC}=\frac{{\text{SW}}_{\mathrm{fresh}}-{\text{SW}}_{\mathrm{dry}}}{{\text{SW}}_{\mathrm{dry}}}$$

which often shows a significant effect on many traits (Luo et al., 2016). On the other hand, we analyzed stoichiometric factors content of soil sample such as ammonium nitrogen (AN), nitrate nitrogen (NN), rapidly available phosphorus (RAP), and total nitrogen (TN) by CleverChem 2000 (Dechem‐Tech Inc.). These nutrient factors are proved to have closely related to leaf nutrient content (R010, Laughlin, 2011). We also measured the pH of soil by acidometer (PB‐100, Sartorius Inc.). Besides, we calculated mean annual temperature (MAT) based on Tang & Fang (2004) and collected mean annual precipitation (MAP) and air humidity (AH) from weather station and WorldClim database. All environment factors’ data were taken the average of three replicates per plot so that it could be used to community analysis.

### 2.3 Constructing phylogenetic tree

Before phylogeny analysis, a super phylogenetic tree should be constructed. To achieve this, a fine species pool size should be determined. The size of local community relative to the regional pool strongly influences statistical power. Too large or too small pool size may affect the power to detect ecological processes (Kraft et al., 2007). According to previous study, the acceptable power of phylogenetic analysis occurs when the community size is 30%–60% relative to regional pool size (Kraft et al., 2007). In our study, 148 woody species was recorded; coincidentally, there were 389 woody species recorded in literature (Ren, 2006), it was a fine size to be a regional species pool (the same method was applied at Webb, 2000). In total, 389 woody species were constructed a super phylogenetic tree using Phylomatic (available at http://phylodiversity.net/). We also structured several different scale species pools (three smaller species pools of 750 m altitudinal wide, even 40 smallest pools for each plot) for testing the fitness of phylogenetic tree, whereas the result of phylogenetic structure calculated by these different scale pools does not show a significant different; on the other hand, species pool which including too little species may lead to incorrect calculations because the overdispersion may be overstated (Kraft et al., 2007). Thus, we decided to use the 389 species’ super tree as a fine‐scale phylogenetic tree. We used Angiosperm Phylogeny Group's APG III consensus tree as a backbone to add species from regional pool based on their taxonomy (Webb & Donoghue, 2005; Chai et al., 2016). BLADJ algorithm was applied to add the evolutionary branch length based on the node date estimated by Wikstrom et al. (Wikstrom, 2001). The algorithm was run at Phylocom 3.0 program (Webb et al., 2008).

### 2.4 Plant sampling and traits measurement

We recorded plant height of every woody species individual in plots. Individual height was measured by tape below 2 m, individual height more than 2 m was measured by height indicator. For each woody species in the same plot, we collected 18–20 fully expanded leaves from various directions of individuals as many as possible. Every leaf we collected was scanned and measured leaf area (LA) by ImageJ Pro 6.0.

There should be minimum number of traits meanwhile maximum number of dimension, and the number of selected traits is expected no more than eight as reported by Daniel (Laughlin, 2014). Therefore, eight morphological, chemical, and progenitive traits were chosen that are related to performance reflecting the response to a particular abiotic environment (Yan et al., 2012). With the exception of LA as mentioned above, specific leaf area (SLA) and leaf dry matter content (LDMC) were measured following standard methods (Cornelissen et al., 2003; Yan et al., 2012). These leaf morphological and physiological traits may show clear relationship with environmental conditions (Cavender‐Bares, Kitajima, & Bazzaz, 2004) and they are the most important factors of community differentiation (Lebrija‐Trejos et al., 2010). Leaf nitrogen content (LNC) and leaf carbon content (LCC) were measured by elemental analyzer (EA3000, EuroVector Inc.) using standard methods as well (Cornelissen et al., 2003; Chacón‐Labella, Cruz, Pescador, & Escudero, 2016). Leaf carbon–nitrogen ratio (C:N) was calculated soon afterward. We examined the individual height data per plot and selected the maximum plant height value (*H*_max_) for every species in the same plot to analyze. *H*_max_ is a trait related to shade tolerance and other abiotc factors (Lavorel et al., 2011; Preston, Cornwell, & Denoyer, 2006). According to previous studies, specific leaf area, plant height, and seed mass (SM) are the main dimensions of plant growing and distribution (Weiher et al., 1999); thus, SM was added to this study. Traits with lower plasticity were more accurately predicted by database value (Cordlandwehr et al., 2013), and SM is a relative conserved trait among species along environmental gradients. We obtained SM data by weighing seed specimen preserved in the specimen museum. Missing data about some species seed mass were compensated by literature review or website information (http://data.kew.org/sid/sidsearch.html). All species trait values used to analyze was a mean value of individual occurring at same plot.

### 2.5 Data analysis

Elevation was the main and the most straightforward gradient in our study system. Apart from elevation, other factors might have great effort for community assembly as well. To explore the relationship among all factors, we applied a correlation analysis and found some factors were covariant (Table 1), and it might create difficulties to find a regular pattern of assembly processes along gradients. Thus, variance partitioning was used to identify the relative roles of all environment factors we selected on traits and phylogeny variation across plots (Pakeman et al., 2009) and found out the most important factor influencing community assembly. Variance partitioning is executed via package “hier.part” running in R 3.1.1 (Qian, Field, Zhang, Zhang, & Chen, 2016). We used mean nearest phylogenetic taxon distance (MNTD) and community‐weighted trait means (CWM) to be inputted data for variance partitioning, representing given community that responded to environment changing. MNTD refers to the average branch length to the nearest co‐occurring taxon (Kraft et al., 2007), it was calculated by Phylocom 3.0 “COMSTRUCT” module with super phylogenetic tree we constructed before, and observed species information of each plot. Note that species data should be arranged into a fine format so that phylocom could be run. The CMW for each trait was calculated as average value of every species of each community weighted by its importance value. Actually, the standard method for calculating CMW is weighted by relative abundance (Garnier et al., 2004; Violle, Lecoeur, & Navas, 2007). Nevertheless in our study, two growth forms that tree and shrub were included, species with greater abundance might not have greater ecological importance. The importance value of each species in each community was calculated as the sum of its relative abundance, relative height, and relative coverage and then divided by 3.

In order to further simplify the environment factors available for gradient analysis, combining the result of variance partitioning (Table 2), a principal component analysis (PCA) was used to reduce the number of dimension of less important environment factors (Qian et al., 2014; ). PCA supplied two orthogonal axes related to some factors severally as comprehensive gradients (Table 3, see Fig. S1 in Appendix S1).

**Table 2:** Results of variance partitioning

Factors	MNTD	LA	SLA	LDMC	*H*_max_	LNC	LCC	C:N	SM
Elevation	21.345^a^	39.302^b^	17.255	24.894^a^	49.246^b^	9.031	29.862^b^	27.153^a^	37.017^b^
SWC	10.480	28.341^a^	34.049^b^	12.809	16.289^a^	10.183	13.426	13.131	29.765^a^
TN	4.446	3.268	5.345	4.288	2.762	5.979	5.212	3.449	3.836
AN	4.493	3.255	5.824	4.140	2.745	6.860	4.612	3.377	3.716
NN	11.498	0.781	3.621	0.567	8.894	6.188	9.902	0.522	0.757
RAP	6.011	11.498	5.177	10.440	7.728	2.675	13.510	2.803	6.700
pH	4.279	7.354	5.492	2.133	8.205	14.931^a^	20.596^a^	2.812	9.179
Slope	4.403	5.511	22.426^a^	40.032^b^	1.450	39.450^b^	1.582	27.330^b^	7.052
WCD	33.045^b^	0.691	0.814	0.697	2.681	4.703	1.297	19.425	1.978

Environment factor codes are as follows: elevation, altitude of plot; SWC, soil water content; TN, soil total nitrogen content; AN, soil ammonium nitrogen content; NN, soil nitrate nitrogen content; RAP, soil rapid available phosphorus content; pH, soil pH value; slope, slope of plot; WCD, woody species coverage degree. Phylogeny and trait codes are as follows: MNTD, mean nearest phylogenetic taxon distance; LA, leaf area; SLA, specific leaf area; LDMC, leaf dry matter content; *H*_max_, maximum plant height; LNC, leaf nitrogen content; LCC, leaf carbon content; C:N, carbon–nitrogen ratio of leaves; SM, seed mass.

a The environment factor has the second best explanatory power for this trait/phylogeny.

b The environment factor has the best explanatory power for this trait/phylogeny.

**Table 3:** Principal component analysis loadings of each environment factor on PCA 1 axis and PCA 2 axis

Factors	PC1	PC2
SWC	−**0.744**	0.307
TN	−**0.905**	−0.076
AN	−**0.922**	−0.137
NN	0.444	**0.642**
RAP	−**0.756**	0.224
PH	0.087	**0.911**
Slope	0.404	−0.360
WCD	−0.237	0.073

Factors that significantly correlated with PCA axes are highlighted in bold.

**Figure 2:**
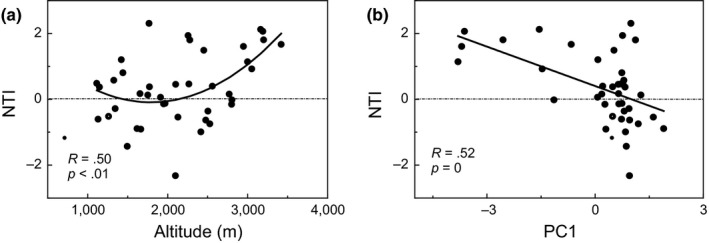
Phylogenetic structure change along environmental gradients. NTI is nearest taxon index. PC1 is the scores of plots on PCA 1 axis. The dotted line represents null expectation

We calculated nearest taxon index (NTI) for indicator about community phylogenetic structure. In general, when the pool size is relatively large, NTI has more power to reveal the assembly pattern (Kraft et al., 2007) than other index. NTI is derived from the MNTD, calculated by subtracting the null model MNTD mean from the observed MNTD value, dividing by the standard deviation of null model trials, and then multiplying by −1.0 (Webb, 2000; Kembel & Hubbell, 2006). Positive values of NTI indicated phylogenetic clustering whereas negative values mean phylogenetic overdispersion (Kraft et al., 2007). NTI was calculated by “COMSTRUCT” module of Phylocom 3.0 (Webb et al., 2008) .The relationship between phylogenetic structure and environmental gradients was discussed for explaining the phylogenetic‐based assembly process along environmental gradients (Figure 2).

As similar as phylogenetic structure, we also investigated the functional traits structure by “COMTRAIT” module of Phylocom 3.0. This algorithm provided an index named standardized effect size of the trait dispersion metric (SES‐metric) to estimate traits dispersion range compared null model. In contrast to NTI, positive value means functional overdispersion, while negative value suggested functional clustering (Webb et al., 2008). Before calculating, trait‐based super trees were constructed by aggregating all this trait values we measured for each trait. After that, the trait mean values of species each plot were required to compare with null model created by the super tree 999 times and outputted values of SES‐metric. Each functional structure was repeated as the method above. Note that the data should be weighted during calculation as nonequivalent for every species in community assembly. We exploring the relationship between each functional structure and environmental gradients and analyzing the trait dispersion patterns along environmental gradients.

Phylogenetic signal is used to infer drivers of community assembly (Burns & Strauss, 2012). It has been increasingly applied in ecological and evolutionary research area (Münkemüller et al., 2012). There are several index to reflect phylogenetic signal, and Blomberg's *K* is the usual and useful index to capture the effect of trait evolution (Münkemüller et al., 2012). We calculated Blomberg's *K* as phylogenetic signal by the approach reported by Münkemüller et al. (2012). The significance of *K* (*p*‐value) was calculated by comparing to null distribution (Yang et al., 2014). Computational process was worked by “phytools” package running in R 3.1.1. *K* = 0 indicates no phylogenetic signal, in other words, trait performs a random pattern comparing phylogeny. *K* = 1 suggests that the trait distribution perfectly conforms to Brownian Motion, and *K* > 1 indicates stronger similarities among closely related species than expected (Liu et al. 2015). In this study, we analyzed the phylogenetic signal of each trait along environmental gradients to detect the trait‐phylogeny relationship with changing environment.

## 3 RESULTS

### 3.1 Main factors of influencing traits and phylogeny variation

Several environment factors had correlation between each other (Table 1). Because MAT was calculated based on elevation (a totally liner relationship), and MAP and AH information which we download from WorldClim or collected from weather station was poorly accuracy, we did not analyze them in the following analysis. Five factors showed significant covariant pattern with elevation except NN, slope, and WCD. Further PCA confirmed this pattern (Table 3, Figure S3). The result of variance partitioning (See Table 2) indicated that elevation could explain the most variation of LA, *H*_max_, LCC, and SM, across plots. Meanwhile, variation of LDMC and C:N could be explained efficiently relatively by elevation as well. Elevation was not the best predictor for phylogenetic variation of interplots, and it could explain 21.345% of total phylogenetic variation while WCD could explain 33.045%. Even so, elevation remained an important effect factor for phylogenetic variation as it had the second highest explaining power. Slope had the best power to explain variation of LDMC, LNC, and C:N. Variation of SLA could be best explained by SWC. Soil nutrient factors often showed poor power to explain both trait and phylogenetic variation among plots.

**Figure 3:**
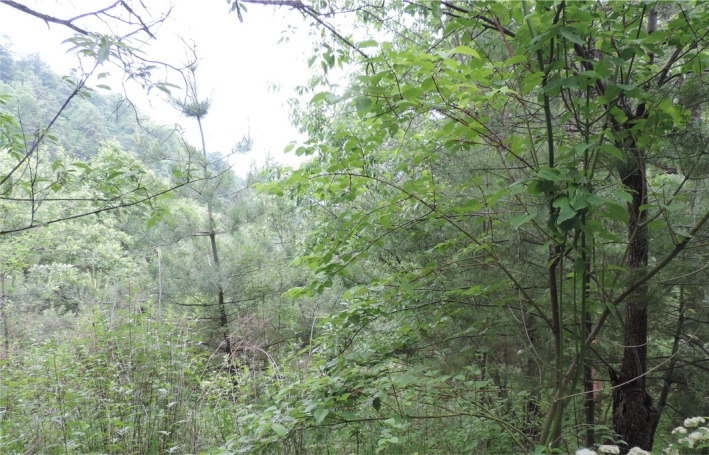
Vegetation in Taibai Mountain

### 3.2 Phylogenetic and functional structure patterns along environmental gradients

NTI exhibited roughly monotonic increasing tendency with increasing altitude. Communities at middle‐ and low‐altitude region (altitude <3,000 m) were difficult to define their phylogenetic structure whether clustering or overdispersion in a comprehensive synthesis as it performed almost the same number of plots below or above 0 value of NTI (17 plots had positive values vs. 16 plots had negative values). However, significant clustering pattern was exhibited at high‐altitude region (altitude ≥3,000 m) on NTI (Figure 2a).

**Figure 4:**
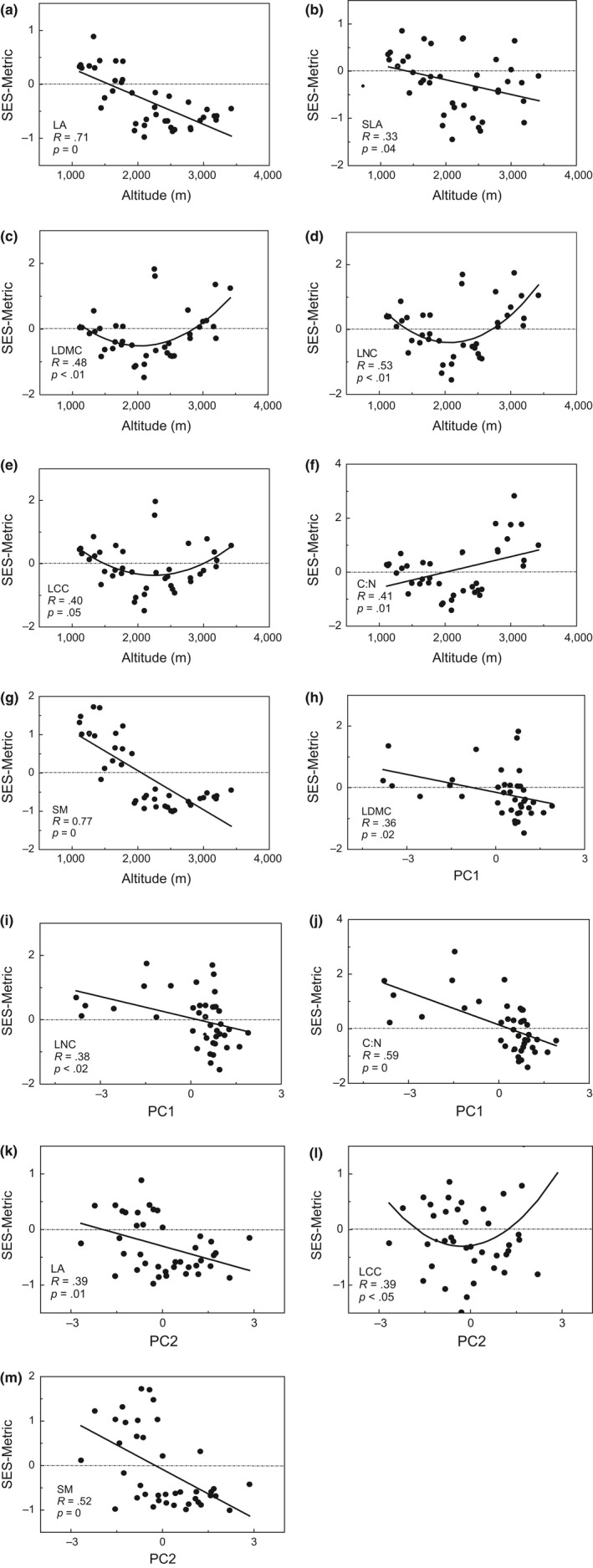
Functional structure change along environmental gradients. SES‐metric is standardized effect size of the trait dispersion metric. Trait codes are as follows: LA, leaf area; SLA, specific leaf area; LDMC, leaf dry matter content; LNC, leaf nitrogen content; LCC, leaf carbon content; C:N, carbon–nitrogen ratio of leaves; SM, seed mass. PC1 and PC2 are the scores of plots on PCA 1 axis and PCA 2 axis. The dotted line represents null expectation

Functional trait structure along elevation showed several patterns. Functional structures of LA and SM showed a decreasing tendency with increasing altitude which meant trait would more clustering along elevation gradient (Figure 4a, g). At relative low‐altitude region (about 2,000 m), LA and SM structures had presented clustering for all plots. SLA also showed a gradual clustering pattern with elevation rising, whereas it did not exhibit a significant clustering at high altitude as LA and SM performed (Figure 4b). LDMC, LNC, and LCC showed similar patterns that their functional structures exhibited a U shape along elevation. For LDMC, LNC, and LCC, communities located at low and high altitude tended to overdispersed than null model, but the most trait‐based overdispersion occurred at middle‐altitude region (about 2,250 m, TBM19 and 20, see Table S1 in Appendix S1) for LDMC and LCC (Figure 4c–e). As a composite trait calculated by LNC and LCC, C:N showed a straightforward overdispersed tendency along elevation (Figure 4f). There was no obvious tendency about relationship between *H*_max_ structure and elevation (ANOVA *p *>* *.05, not shown in graph).

**Figure 5:**
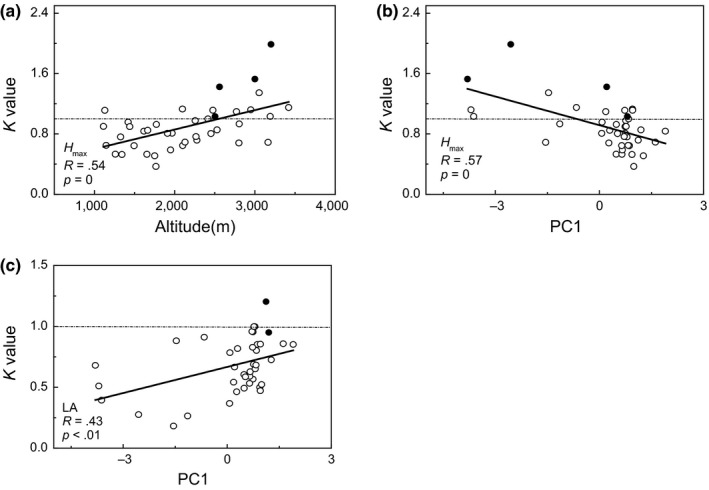
Phylogenetic signal change along gradients. *K* value is the value of Blomberg's *K* for each trait in every community. PC1 is the scores of plots on PCA 1 axis. H_max_ is maximum plant height, LA is leaf area. Solid cycle in graph means a phylogenetic signal with statistical significance. The dotted line represents Brownian Motion

PCA axis 1 (PC1) could explain 40.23% of total variance of eight environment factors except elevation, and PCA axis 2 (PC2) could explain 19.33% of it (see Fig. S1 in Appendix S1). Only two axes were found statistical significance. PC1 inversely associated with AN, TN, RAP, and SWC significantly. PC2 positively correlated with pH and NN (Table 3). Scores on PC1 and PC2 for each plot were used as compound environment gradients. For PC1 gradient, NTI showed overdispersed tendency with score increasing on PC1, whereas LDMC, LNC, and C:N showed clustering tendency (Figures 2b, 4h, 5i, j). Other trait structures had no relationship with PC1 (not shown in graph).For PC2 gradient, SLA and SM clustered with PC2 score increasing (Figure 4k, l). Besides, LCC exhibited a U‐shaped pattern along PC2 gradient, seemed like the pattern which was shown above (Figure 4m). In PC1 and PC2, WCD was neglected relatively (Table 3). We also detected the relationship between WCD and phylogenetic and functional structures but found no relationship (ANOVA *p *>* *.05, not shown in graph).

### 3.3 Phylogenetic signal change along environment gradients

Blomberg's *K* values of each trait were showed at Table S2 in Appendix S1. LDMC showed a relatively stronger phylogenetic signal as it had the most plots that the *K* value >1 (18 plots out 40). Also, LDMC had the most number of that K value with statistical significance (14 plots out 40). However, only Blomberg's *K* of Hmax exhibited palpable shift along elevation gradient. Blomberg's *K* of *H*_max_ increased with increasing altitude and suggested there would be stronger phylogenetic signal of *H*_max_ at higher altitude (Figure 5a). In contrast, phylogenetic signal of Hmax decreased along PC1 gradient (Figure 5b). Blomberg's *K* of SLA increased along PC1 gradient, but most of the *K* value <1, suggested that there was only a tendency that the distribution of SLA would be more similar with phylogeny along PC1 gradient (Figure 5c). Besides, none of traits showed variance regular trend with PC2.

## 4 DISCUSSION

### 4.1 Elevation is the main factor driving the shift of community assembly pattern

Community structure varies along elevational gradients commonly (Sundqvist, Sanders, & Wardle, 2013). In our study, elevation could explain efficiently most of trait variations. Although slope could best explain some trait variation as well, it seemed to lead to this appearance by accident. Variation of slope for each plot was limited; moreover, we obtained slope of each plot by measuring the slope in central point of plots, and it merely reflected a general situation of sites. Actually, there were a series of heterogeneous subhabitats in the same plot. Howbeit, there are evidences that LDMC related with locality conditions (Lavorel et al., 2011) as well, perhaps caused by water availability which related with topographic factors. On the other hands, elevation had the second best power to explain LDMC and C:N variation. Meanwhile, SWC had the best power to explain variation of SLA as it reported by literature (Ackerly & Cornwell, 2007). It was important to note that SWC had a strong correlation with elevation in our study (correlation coefficient = 0.895, *p *<* *.01, Table 1), because the middle‐altitude region had relative more precipitation while the high‐altitude region tended to be swampiness and then soil moisture increased. For phylogenetic variation, elevation could explain 21.3% variation, remained an important factor as a predictor. WCD was the only biotic environment for our gradient analysis and showed the best power to explain phylogenetic variation, perhaps it caused by competition of coexisting species.

Accordingly, elevation is the main factor leading to variation of traits and phylogeny, which drove community assembly processes. This conclusion is coincide with the study in southeastern China (Legendre et al., 2009) but contrary to Kooyman's study, which found elevation accounted for little of the variation in community phylogeny and trait. However, Kooyman's study experimented in tropical forest and the highest altitude in their study was only 1,650 m (Kooyman, Rossetto, Allen, & Cornwell, 2012).

Although elevation may influence ambient humidity, total radiation, precipitation, wind velocity, seasonality, geological substrates, soil formation processes, and disturbance history (Pickett 1989, Körner 2007), based on our data, we only found a significant relation between elevation and temperature, which was consistent with Körner (2007). Indubitability, there should be a lot of other factors we not measured, leading to a limit to clarify which factor would directly influence community assembly processes. Thus, although elevation may affect community assembly directly, we would rather see elevation as a complex environmental factor, even as a main factor driving the shift of community assembly pattern.

### 4.2 Phylogenetic and several functional structures cluster in alpine area: evidence for environmental filtering

Phylogenetic structure showed an obvious clustering tendency at high‐altitude region (above 3,000 m) in our study. For traits analysis, functional structure of LA, SLA, and SM which reflect plant growth and reproduction (Yan et al., 2012), exhibited the similar pattern with phylogeny. These results indicated the forceful filtering process influenced community assembly in alpine area with limited hydrothermal resources. These results are consistent with previous studies (Spasojevic, Yablon, Oberle, & Myers, 2014; Mori et al., 2013). Based on niche theory which emphasizing deterministic processes in community assembly (Helsen, Hermy, & Honnay, 2012), species colonizing a site with a particular set of environmental conditions will tend to exhibit similarity for certain phenotypic traits and leading to trait convergence (Weiher & Keddy, 1998). Besides, Mayfield & Levine (2010) reported competition can sometimes lead to clustering pattern as well. In consideration of the extreme conditions (for instance, low temperature, high UV‐B radiation, poorly developed soil, and strong wind)in alpine region, and then, these extreme conditions filter are more likely to some traits (such as LA, SLA, and SM showing in this study) or taxa which fitting the harsh situation into a similar phenotype.

However, a surprising phenomenon exhibited by LDMC, LNC, and LCC which showed gradually overdispersed tendency at high‐altitude region while clustered at middle‐altitude region. In our study area, 3,000 m or so is the turning point that significant changes would occur in vegetation type. *Betula utilis* forest which refers to latifoliate angiosperm dominated would transform to *Abies fargesii* forest which dominated by needle‐leaved gymnosperm at that altitudes. There were significant differences on LDMC, LNC, and LCC between angiosperm and gymnosperm in our analysis, leading to a wide variation range of these traits in alpine area where community species is composed of both plant types. On the other point of view, these traits are conserved. Hence, the phenomena were consistent with our functional structure analysis based on LDMC, LNC, and LCC in alpine area. Besides, abnormal overdispersed phenomena occurring at middle‐altitude region in LDMC, LNC, and LCC might be caused by the similar circs as those communities were broad‐leaved forest mixed by *Pinus armandii*.

### 4.3 Community assembly pattern in middle‐ and low‐altitude region

Phylogenetic structure at middle‐ and low‐altitude showed low resolution and poor sensitive to explore a characteristic assembly process. Processes acting in opposing directions, such as limiting similarity and filtering, may act to remove any structure detectable with statistics (Kraft et al., 2007). In contrast, we found a obvious evidence on most trait‐based gradient analysis that trait showed a convergent tendency with altitude increasing within middle‐ and low‐altitude region (below 3,000 m), indicating traits filtering at higher altitude.

In general, middle‐altitude mountain area has the best hydrothermal conditions and the highest productivity, seems to be dominated by species interaction then leading to limiting similarity and showing overdispersed patterns of traits. Actually, traits filtering may not necessarily be the result of abiotic filtering under harsh conditions but could likely also result from biotic interactions in productive habitats (Bernard‐Verdier et al., 2012). In other words, functional convergence in community may due to competition‐sorting species with different competitive abilities and not only environmental filtering as commonly assumed (Kunstler et al., 2012; Santoro, Jucker, Carboni, & Acosta, 2012). Hence, traits convergence in middle‐altitude region was probably contributed by filtering on competitive abilities, the other trait for plant colonization, but a result of interspecific competition (Swenson et al. 2011). However, the relative role of environmental filtering and species competitive exclusion was difficult to distinguish, merely suggested deterministic process dominated community assembly based on functional traits.

### 4.4 Phylogenetic‐based and trait‐based assembly along soil gradients

Both PC1 and PC2 were correlated closely with soil factors (AA, TN, RAP, SWC for PC1 and pH, NN for PC2), and suggested PC1 and PC2 probably could be substitutes for soil factors. Owing to the negative correlation between PC1 and AA, TN, RAP, and SWC, higher score on PC1 meant lower value of those soil factors. With PC1 score decreasing, phylogenetic structure showed more and more convergent. Remember that AA, TN, RAP, and SWC also had a significant positive correlation with elevation (Table 1), thus lower PC1 score probably meant higher altitude, leading to a clustering pattern. As PC1 had an indirect relationship with elevation, functional structures along PC1 were deemed to have similar patterns that they exhibited along elevation. However, apart from C:N, the fantastic U‐shaped regularities of LDMC and LNC along elevation gradient did not reappear in PC1 gradient. In contrast, for LDMC and LNC, more convergent tendency was shown along score on PC1 linearly, indicated filtering process occurred gradually with lower nutrients and water availability. Soil nutrient content and SWC may influence LNC and LDMC (Heineman et al., 2016), therefore soil factors related to PC1 would influence functional assembly based on LNC and LDMC. Besides, the different performance of phylogeny and functional traits along PC1 gradient may suggest community structure is not explained by soil fertility directly (Laliberté, Zemunik, & Turner, 2014).

For PC2, the similar pattern compared to elevation was shown on LA, LCC, and SM, suggested an interior relation between PC2 and elevation. The most correlated factor for PC2 was pH, which had a significant positive correlation with elevation. Structure of the other traits and phylogeny showed no relationship with PC2 and indicated there were no effect for phylogenetic or more functional assembly along this gradient.

### 4.5 Phylogenetic signal shift along environmental gradients

Phylogenetic signal detected the performance of phylogeny and traits whether coherent. If phylogeny was inconsistent with trait, the trait may not be a phylogenetically conserved trait (Satdichanh et al., 2015; Pillar & Duarte 2010). LDMC showed more phylogenetic signal than other traits. In discussion of relationship between LDMC structure and elevation, it was proved LDMC seemed to be a phylogenetically conserved trait. However, the other conserved traits we considered such as LNC and LCC showed significant phylogenetic signal hardly. For the other traits, phylogenetic signal with statistical significant was not too much. Phenotypic plasticity resulting in trait divergence may decrease phylogenetic signal in community assembly (Burns & Strauss, 2012), in previous study, intra‐specific phenotypic variance could lead to 30% of total traits variance (Albert et al., 2010), whereas there were still other authors argued that intra‐specific shifts played a small role (Cornwell & Ackerly, 2009). However, the intra‐specific variance was not factored into the algorithm of Blomberg's *K*.

Actually, the phylogenetic signal we calculated in this study was based on each community, rather than the synthesized signal among all communities. Therefore, traits in different community might have different phylogenetic signal. Undeniably, the system scale for assessing phylogenetic signal seemed too small, it might decrease the power to detect phylogenetic signal. However, we analyzed each phylogenetic signal for each trait along environmental gradients, aimed to find the variance tendency. In such small scale, LCC showed significant phylogenetic signal and suggested LCC may be the most sensitive factor to explore the relationship of traits and phylogeny. Besides, in our study, only *H*_max_ which related shade tolerance and light recourse competition ability (Preston et al., 2006) showed a significant shift along elevation gradient, suggested at higher elevation, plant maximum height tended to be a closer relation to phylogeny, resulting from environmental filtering probably. Because of the indirect correlation between elevation and PC1, *H*_max_ showed similar relationship with PC1. There was no significant phylogenetic signal change for the other traits, indicated these traits’ phylogenetic signal did not change along environmental gradients.

### 4.6 Further works for understanding community assembly

Phylogenetic‐based and trait‐based methods have respective merits to explore community assembly processes. For these ecological processes, performances of phylogeny and functional traits may be independent of each other (Yang et al., 2012). Several previous studies indicated that different assembly processes are detected based on two different methods, even in the same community (Zhang et al., 2015; Liu, Wang et al., 2013; Liu, Swenson, Zhang, & Ma, 2013; Purschke et al. 2013; Swenson et al., 2012). Meanwhile, the assembly pattern merely performed on phylogenetic‐based or trait‐based method seems lack of further evidence. For instance, phylogenetic clustering may result from competitive exclusion (Mayfield & Levine, 2010), whereas phylogenetic overdispersion has several possible explanations (Kembel & Hubbell, 2006). Besides, there are different assembly patterns detected by different traits. Hence, it is hardly to draw a conclusion based on one method. Combining phylogenetic‐based and trait‐based methods along environmental gradients can provide a comprehensive insight into community assembly dynamic (Lortie et al., 2004; Mcgill et al., 2006), which is crucial for understanding how communities will behave under environment change (Gotzenberger et al., 2012).

We need more works on detecting assembly processes using phylogenetic‐based and trait‐based methods, especially using “correct” methods. In our study, the trait values for calculating functional structures were weighted that was dissimilar with previous studies. Plants occurring at communities have great difference on their abundance or importance values, means they are ecologically nonequivalent. For this reason, traits should be weighted by their relative abundance or importance value so that it can reveal communities’ status rather than species’. However, for phylogenetic‐based method, weighting is not necessary, because phylogeny represents the relative relationship of pairwise species, which reflects the community's structure. Actually, a perfect system for understanding community assembly should base on individual in community instead of species. We merely use a compromising way for assessing community assembly in this study, and a more effective way to combine the two useful methods needs further works.

Meanwhile, phylogenetic signal will be a useful way to explore traits evolution along environmental gradients. In our study, there were only two traits showed detectable change along gradients, and researchers should test more traits and try to find out relationships of traits plasticity and phylogeny along environment change, which result from assembly processes, that may provide another perspective to understand community assembly.

## CONFLICT OF INTEREST

None declared.

## AUTHOR'S CONTRIBUTIONS

J.S.X. conducted field work, analyzed data, and wrote the article. Y.C. helped completing major field work. M.Y. and Y.X.G. designed the study. Y.F.C. and M.W. suggested approaches for data analysis. T.L. provided most extra seed mass data. L.X.Z. and C.G.Z. participated in laboratory experiment, and X.L. conducted article writing.
