# Supplementary material for: Using phylogeny and functional traits for assessing community assembly along environmental gradients: A deterministic process driven by elevation
Source: Ecol Evol. 2017 Jun 2;7(14):5056–69. doi: 10.1002/ece3.3068 (PMC5528205; doi:10.1002/ece3.3068)
Supplement: Supplementary file 1 [file ECE3-7-5056-s001.doc]

**Table S1** The information of plots.

| Plots | Altitude (m) | Longitude (°) | Latitude(°) | Dominant species |
| --- | --- | --- | --- | --- |
| TBM001 | 1113 | 107.2081 | 34.0892 | *Quercus aliena* var*. acutiserrata* |
| TBM002 | 1130 | 107.7083 | 34.0892 | *Quercus aliena* var*. acutiserrata* |
| TBM003 | 1147 | 107.7086 | 34.0892 | *Quercus aliena* var*. acutiserrata* |
| TBM004 | 1261 | 107.6992 | 34.0806 | *Quercus aliena* var*. acutiserrata* |
| TBM005 | 1326 | 107.6972 | 34.0808 | *Quercus aliena* var*. acutiserrata* |
| TBM006 | 1344 | 107.6964 | 34.0806 | *Quercus aliena* var*. acutiserrata* |
| TBM007 | 1420 | 107.6944 | 34.0789 | *Quercus aliena* var*. acutiserrata* |
| TBM008 | 1440 | 107.6933 | 34.0781 | *Quercus aliena* var*. acutiserrata* |
| TBM009 | 1494 | 107.6917 | 34.0775 | *Quercus aliena* var*. acutiserrata* |
| TBM010 | 1617 | 107.6925 | 34.0722 | *Quercus aliena* var*. acutiserrata* |
| TBM011 | 1654 | 107.6939 | 34.0050 | *Quercus aliena* var*. acutiserrata* |
| TBM012 | 1665 | 107.6936 | 34.0706 | *Quercus aliena* var*. acutiserrata* |
| TBM013 | 1751 | 107.6944 | 34.0683 | *Quercus aliena* var*. acutiserrata* |
| TBM014 | 1767 | 107.6950 | 34.0672 | *Quercus aliena* var*. acutiserrata* |
| TBM015 | 1773 | 107.6953 | 34.0667 | *Quercus aliena* var*. acutiserrata* |
| TBM016 | 1911 | 107.6947 | 34.0597 | *Quercus wutaishanica* |
| TBM017 | 1950 | 107.6944 | 34.0581 | *Quercus wutaishanica* |
| TBM018 | 1968 | 107.6944 | 34.1581 | *Quercus wutaishanica* |
| TBM019 | 2097 | 107.6972 | 34.0567 | *Quercus wutaishanica* |
| TBM020 | 2101 | 107.6944 | 34.0567 | *Quercus wutaishanica* |
| TBM021 | 2129 | 107.6981 | 34.0564 | *Quercus wutaishanica* |
| TBM022 | 2255 | 107.7008 | 34.0517 | *Salix cathayana* & *Betula albo-sinensis* |
| TBM023 | 2265 | 107.7011 | 34.0522 | *Salix cathayana* & *Betula albo-sinensis* |
| TBM024 | 2277 | 107.7017 | 34.0514 | *Betula albo-sinensis* |
| TBM025 | 2415 | 107.7058 | 34.0481 | *Rhododendron purdomii* & *Betula albo-sinensis* |
| TBM026 | 2452 | 107.7056 | 34.0478 | *Betula albo-sinensis* |
| TBM027 | 2477 | 107.6875 | 34.0456 | *Corylus ferox* & *Betula albo-sinensis* |
| TBM028 | 2503 | 107.7103 | 34.0453 | *Rhododendron purdomii* & *Betula albo-sinensis* |
| TBM029 | 2528 | 107.7100 | 34.0456 | *Betula albo-sinensis* |
| TBM030 | 2559 | 107.7097 | 34.0456 | *Betula albo-sinensis* |
| TBM031 | 2770 | 107.7153 | 34.0439 | *Betula albo-sinensis* |
| TBM032 | 2800 | 107.7158 | 34.0439 | *Betula utilis* |
| TBM033 | 2804 | 107.7156 | 34.0433 | *Betula utilis* |
| TBM034 | 2948 | 107.8092 | 34.0094 | *Abies fargesii* |
| TBM035 | 3000 | 107.8094 | 34.0081 | *Abies fargesii* |
| TBM036 | 3053 | 170.4764 | 34.0044 | *Abies fargesii* |
| TBM037 | 3163 | 107.8100 | 34.0042 | *Larix chinensis* |
| TBM038 | 3191 | 107.8094 | 34.0028 | *Larix chinensis* |
| TBM039 | 3200 | 107.8097 | 34.0039 | *Larix chinensis* |
| TBM040 | 3480 | 107.8059 | 33.9967 | *Rhododendron capitatum* |

**Table S2** Phylogenetic signal of each trait in every community. The value is Blomberg's K. Trait codes are as follows: LA, leaf area; SLA, specific leaf area; LDMC, leaf dry matter content; H_max_, maximum plant height; LNC, leaf nitrogen content; LCC, leaf carbon content; C:N, carbon nitrogen ratio of leaves; SM, seed mass. Significant phylogenetic signals are highlighted in bold.

| Plots | LA | SLA | LDMC | H_max_ | LNC | LCC | C.N | SM |
| --- | --- | --- | --- | --- | --- | --- | --- | --- |
| TBM01 | 0.605 | 0.592 | 0.382 | 0.531 | 0.439 | 0.256 | 0.524 | 0.966 |
| TBM02 | 0.998 | 0.893 | 0.980 | 0.760 | 0.878 | 0.732 | 0.923 | 1.428 |
| TBM03 | 0.499 | 0.840 | 0.784 | 0.527 | 0.794 | 0.819 | 0.614 | 0.643 |
| TBM04 | 0.785 | 0.685 | 1.286 | 0.955 | 0.679 | 0.851 | 0.651 | 0.799 |
| TBM05 | 0.566 | 0.812 | 0.647 | 0.896 | 0.649 | 0.385 | 0.703 | 0.691 |
| TBM06 | 0.851 | 0.826 | **1.150** | 0.643 | 0.878 | 0.763 | 1.053 | 0.609 |
| TBM07 | 0.851 | 0.530 | 0.799 | 0.837 | 0.504 | **1.490** | 1.088 | 0.590 |
| TBM08 | 0.590 | 0.598 | **1.034** | 0.529 | 0.777 | 0.829 | 0.671 | 0.840 |
| TBM09 | 0.818 | 0.775 | 1.131 | 0.848 | 0.706 | **1.542** | 0.712 | 0.888 |
| TBM10 | 0.725 | **1.030** | **1.116** | 0.509 | 0.633 | 0.812 | 0.986 | 0.583 |
| TBM11 | 0.523 | 0.364 | 0.420 | 0.368 | 0.692 | 0.560 | **0.882** | 0.668 |
| TBM12 | 0.492 | 0.458 | 0.783 | 0.925 | 0.681 | 0.671 | 0.359 | 0.406 |
| TBM13 | 0.628 | **1.253** | **1.071** | 0.587 | **1.138** | 0.630 | **1.082** | 0.747 |
| TBM14 | 0.955 | 0.971 | **1.258** | 0.809 | 0.894 | 0.732 | 0.806 | 0.707 |
| TBM15 | 0.367 | 0.586 | 0.623 | 0.808 | **0.815** | 0.357 | 0.660 | 0.788 |
| TBM16 | 0.471 | 0.489 | 0.727 | 1.130 | 0.693 | 0.465 | 1.007 | 0.742 |
| TBM17 | 0.532 | 0.551 | **1.173** | 0.644 | 0.415 | 0.794 | 0.879 | 0.833 |
| TBM18 | 0.858 | 0.366 | 0.849 | 0.690 | 0.773 | **1.111** | 1.246 | 0.700 |
| TBM19 | **1.203** | 0.937 | **1.463** | 0.716 | 0.963 | 0.614 | 1.359 | **1.882** |
| TBM20 | 0.958 | 0.848 | 1.106 | 0.764 | 1.028 | 0.820 | 1.289 | 0.670 |
| TBM21 | 0.689 | 0.262 | 0.396 | 0.976 | 0.667 | 0.511 | 1.285 | **2.194** |
| TBM22 | 0.585 | 0.364 | **1.925** | 0.805 | 0.536 | 0.547 | **1.537** | 0.498 |
| TBM23 | 0.803 | 0.722 | **1.208** | 0.999 | 0.938 | 0.652 | 1.358 | 0.886 |
| TBM24 | 0.854 | 0.967 | 1.106 | 1.113 | 0.918 | 1.052 | 1.035 | 0.743 |
| TBM25 | 0.650 | 0.294 | **0.886** | **1.031** | 0.901 | 0.537 | 0.787 | **2.239** |
| TBM26 | **0.950** | 0.339 | 0.537 | 0.854 | 0.501 | 0.663 | 0.481 | 0.693 |
| TBM27 | 0.666 | 0.403 | **1.300** | **1.424** | **1.618** | 0.758 | **2.478** | **1.358** |
| TBM28 | 0.542 | 0.493 | **1.248** | 1.096 | 0.840 | 0.251 | **1.872** | 0.328 |
| TBM29 | 0.462 | **1.129** | 0.769 | 0.680 | **1.131** | 0.934 | **1.809** | 0.803 |
| TBM30 | 0.264 | 0.477 | 0.499 | 0.933 | 0.642 | 0.785 | 1.589 | 0.539 |
| TBM31 | 0.999 | 0.856 | 0.964 | 0.900 | 1.063 | 0.915 | 1.006 | 0.827 |
| TBM32 | 0.828 | 0.931 | **1.251** | 1.112 | 0.815 | 0.877 | 0.831 | 0.782 |
| TBM33 | 0.682 | 1.128 | 1.062 | 0.647 | 0.887 | 0.944 | 1.170 | 1.307 |
| TBM34 | 0.394 | 0.247 | 0.316 | 1.033 | 0.381 | 0.968 | 0.691 | 0.756 |
| TBM35 | 0.276 | **1.156** | 0.168 | **1.988** | 0.606 | 0.232 | 0.608 | 0.780 |
| TBM36 | 0.181 | 0.225 | 0.258 | 0.688 | 0.174 | 0.140 | 0.412 | 0.267 |
| TBM37 | 0.881 | **1.543** | 0.669 | 1.345 | 0.873 | **1.461** | 0.798 | 0.783 |
| TBM38 | 0.680 | **1.389** | **1.361** | **1.527** | **1.437** | 1.097 | **1.664** | 0.769 |
| TBM39 | 0.510 | 0.684 | 0.806 | 1.120 | 0.398 | 0.581 | 0.776 | 0.749 |
| TBM40 | 0.911 | 0.887 | 0.928 | 1.151 | 0.879 | 0.813 | 0.813 | 0.868 |

**Figure S1** PCA loadings plot graph. PC1 and PC2 are PCA 1 axis and PCA 2 axis. Factor codes are as follows: SWC, soil water content; TN, soil total nitrogen content; AN, soil ammonium nitrogen content; NN, soil nitrate nitrogen content; RAP, soil rapid available phosphorus content; pH, soil pH value; Slope: slope of plot; WCD, woody species coverage degree. Length of arrow means this factor's contribution for PCA. Angle between each arrow means the correlation of each factor, an acute angle represents positive correlation, an obtuse angle represents negative correlation, a right angle represents irrelevant. Value outside axis is correlation coefficient for each PCA axis. PC1 can explain 40.23% total environmental variance, PC2 can explain 19.33% total environmental variance.
